# Lymphocytes and Macrophages Are Infected by *Theileria equi*, but T Cells and B Cells Are Not Required to Establish Infection *In Vivo*

**DOI:** 10.1371/journal.pone.0076996

**Published:** 2013-10-07

**Authors:** Joshua D. Ramsay, Massaro W. Ueti, Wendell C. Johnson, Glen A. Scoles, Donald P. Knowles, Robert H. Mealey

**Affiliations:** 1 Department of Veterinary Microbiology & Pathology, Washington State University, Pullman, Washington, United States of America; 2 Animal Disease Research Unit, Agricultural Research Service, USDA, Pullman, Washington, United States of America; University of Minnesota, United States of America

## Abstract

*Theileria equi* has a biphasic life cycle in horses, with a period of intraleukocyte development followed by patent erythrocytic parasitemia that causes acute and sometimes fatal hemolytic disease. Unlike *Theileria spp*. that infect cattle (*Theileria parva* and *Theileria annulata*), the intraleukocyte stage (schizont) of *Theileria equi* does not cause uncontrolled host cell proliferation or other significant pathology. Nevertheless, schizont-infected leukocytes are of interest because of their potential to alter host cell function and because immune responses directed against this stage could halt infection and prevent disease. Based on cellular morphology, *Theileria equi* has been reported to infect lymphocytes *in vivo* and *in vitro*, but the specific phenotype of schizont-infected cells has yet to be defined. To resolve this knowledge gap in *Theileria equi* pathogenesis, peripheral blood mononuclear cells were infected *in vitro* and the phenotype of infected cells determined using flow cytometry and immunofluorescence microscopy. These experiments demonstrated that the host cell range of *Theileria equi* was broader than initially reported and included B lymphocytes, T lymphocytes and monocyte/macrophages. To determine if B and T lymphocytes were required to establish infection *in vivo*, horses affected with severe combined immunodeficiency (SCID), which lack functional B and T lymphocytes, were inoculated with *Theileria equi* sporozoites. SCID horses developed patent erythrocytic parasitemia, indicating that B and T lymphocytes are not necessary to complete the *Theileria equi* life cycle *in vivo*. These findings suggest that the factors mediating *Theileria equi* leukocyte invasion and intracytoplasmic differentiation are common to several leukocyte subsets and are less restricted than for *Theileria annulata* and *Theileria parva*. These data will greatly facilitate future investigation into the relationships between *Theileria equi* leukocyte tropism and pathogenesis, breed susceptibility, and strain virulence.

## Introduction

*Theileria equi* is a tick-transmitted apicomplexan hemoprotozoan parasite that causes acute hemolytic disease (equine piroplasmosis) and persistent infection of wild and domestic equids throughout the world [1], [2]. The life cycle of *Theileria equi* is biphasic in the mammalian host, with a period of intraleukocyte development (pre-erythrocytic schizogony) followed by patent erythrocytic parasitemia [3], [4]. The pre-erythrocytic stage of *T. equi* has not been associated with clinical disease in equids and relatively little work has been done to characterize host-parasite interaction during this phase of infection. *In vitro* and *in vivo*, tick-transmitted *T. equi* sporozoites infect mononuclear leukocytes and differentiate into multinucleated schizonts (schizogony), which further divide to form erythroinvasive merozoites [4]. Based on morphology, schizont-infected cells have been characterized as lymphocytes, but this finding has not been confirmed [3], [4]. Conversely, the leukocyte tropism is very well described for two close relatives of *T. equi*, *Theileria annulata* and *Theileria parva* [5]. The aforementioned *Theileria spp*. are bovine pathogens that infect both leukocytes and erythrocytes; however, unlike *T. equi*, the diseases caused by *T. annulata* (Tropical Theileriosis) and *T. parva* (East Coast Fever) are largely due to the transformation and dissemination schizont-infected leukocytes and lymphoproliferation [6]–[9].

*In vivo*, *T. annulata* sporozoites invade macrophages, and to a lesser extent B lymphocytes [10]–[12], and differentiate into macroschizonts that alter the host cell transcriptome to induce proliferation, dissemination, and modify gene expression [13]–[16]. Native *Bos indicus* cattle (Sahiwal) are significantly more resistant to Tropical Theileriosis than are *Bos taurus* cattle (Holstein) due to their ability to regulate the inflammatory response and limit the dissemination of infected cells [15]–[17]. Broad transcriptome analysis of uninfected and infected Holstein and Sahiwal macrophages identified significant differences in the expression of genes related to inflammation and immune responses, suggesting that the relative resistance of Sahiwal cattle is due to an inherent difference in how the host cell functions following infection [15], [18]. This demonstrates how the tropism of *T. annulata* for macrophages directly impacts the variation in virulence and pathogenesis observed in these two breeds. The specific phenotype of host cells infected by *T. parva* (predominantly T lymphocytes *in vivo* [10], [11], [19]–[21]) is of similar importance in East Coast fever. For example, the virulent Muguga strain sporozoites preferentially infecting CD4^+^ T lymphocytes and the less virulent Chitongo strain only infect and transform CD8^+^ T cells [22].

Prior to this study, the phenotype of *T. equi*-infected leukocytes had only been defined based on cellular morphology, which limits the ability to make meaningful comparisons with other *Theileria spp*., identify correlates with other phenotypic traits (virulence), and investigate how this stage of infection impacts host immunity. Based on the observations of morphologic studies it could be hypothesized that B and T lymphocytes are required to establish infection with *T. equi* sporozoites [4]. This hypothesis was specifically tested in the current study by: 1) immunophenotyping schizont-infected cells *in vitro* with flow cytometry and immunofluorescence antibody microscopy (IFA), and 2) attempting to establish infection in young Arabian horses (foals) with severe combined immunodeficiency (SCID) via sporozoite inoculation. Horses affected with SCID lack functional B and T lymphocytes due to a frameshift mutation in the catalytic subunit of DNA-dependent protein kinase (DNA-PKcs), which results in a complete absence of mature B and T lymphocytes [23]–[25]. Establishing infection in SCID foals with *T. equi* sporozoites would therefore demonstrate whether or not B and T lymphocytes are necessary in the life cycle of *T. equi* within the vertebrate host.

## Materials and Methods

### Ethics Statement

All animal experiments were carried out in strict accordance with the recommendations in the Guide for the Care and Use of Laboratory Animals of the National Institute of Health and in conformance with the United States Department of Agriculture animal research guidelines, under a protocol approved by the Washington State University Institutional Animal Care and Use Committee.

### Horses

Two SCID foals (SCID1 and SCID2), one immunocompetent Arabian foal (Foal1), and 14 adult immunocompetent Arabian or Arabian/pony mixed breed horses (HS1-6, HT1-4, HM1, H1-3; S = sporozoite inoculated, T = tick-transmitted, and M = merozoite inoculated) were used in this study. Foals were approximately one month old at the beginning of the experimental period and all other horses ranged from six months to nine years of age. SCID foals were obtained by selective breeding of Arabian horses (or Arabian/pony crosses) heterozygous for the SCID trait [26]. SCID was initially diagnosed based on persistent lymphopenia and subsequently confirmed by identifying the homozygous mutation in the DNA-PKcs gene sequence [23], [27], [28]. SCID foals were maintained as described previously [28]–[30].

### Tick-transmission, sporozoite isolation, cryopreservation, and IV inoculation

Adult male *R. microplus* ticks were reared and infected with the *T. equi* Florida strain for tick-transmission as previously described [31]. Briefly, approximately 20,000 larvae were fed on a Holstein calf for 14 days at which time engorged nymphs were forcibly removed and allowed to molt to the adult stage in an incubator for three days at 26°C, at 94% relative humidity and a 12-h photoperiod. To acquire *T. equi* infection, 500 adult male *R. microplus* ticks were allowed to feed under a cloth patch for 8 days on horse HM1 during acute infection. HM1 was infected prior to acquisition feeding by intravenous (IV) inoculation with a Florida strain *T. equi* merozoite blood stabilate. Following acquisition feeding, adult male ticks were collected and incubated for 3 days at 15°C, at 94% relative humidity, with a 12-h photoperiod. Approximately 200 male ticks survived incubation and were subsequently applied under a cloth patch in groups of 50 and allowed to feed on HT1-4 for 10 days to complete tick-transmission.

To obtain *T. equi* sporozoites for sporozoite inoculation, salivary gland pairs were dissected from infected *R. microplus* adult male ticks (tick infection as described above). Prior to beginning IV inoculation experiments, the salivary glands from 5–10 cohort ticks were analyzed to determine the infection rate by extracting genomic DNA and performing quantitative real-time PCR targeting a single copy *T. equi* EMA-1 gene, as previously described [31], [32]. Remaining salivary gland pairs were then washed in sterile PBS and crushed in a glass homogenizer to release sporozoites. The homogenate was centrifuged at 300 g for 5 min and the supernatant containing sporozoites was collected. Horses HS1-4 were inoculated IV with ∼1×10^5^–10^6^ *T. equi* sporozoites (as determined by quantitative real-time PCR). Unused salivary glands from the previous tick rearing were washed in 0.5 ml of complete media (RPMI 1640 supplemented with 10% heat inactivated fetal bovine serum, 200 i.u./ml benzyl penicillin, 200 ug/ml streptomycin sulphate, 50 µg/ml gentamycin and 5×10^−5^ M 2-mercaptoethanol) and crushed in a glass homogenizer to release sporozoites. The supernatants were divided into 2×10^6^ sporozoite aliquots in 2 ml cryovials and brought up to 1 ml volume, with RPMI and DMSO (final fluid composition: 45% RPMI, 45% complete media, and 10% DMSO). All vials were frozen at −80°C and transferred to liquid nitrogen for later use. Aliquots of cryopreserved sporozoites were rapidly thawed in a 37°C water bath and inoculated IV, undiluted or at a 1∶10 dilution in sterile PBS, in SCID foals and the immunocompetent control animals (Foal1, HS5-6). In the first experimental inoculation, SCID1, Foal1, HS5 and HS6 were inoculated with cryopreserved tick salivary gland homogenates containing ∼1×10^6^ sporozoites. SCID1 was subsequently re-inoculated with ∼4×10^6^ cryopreserved sporozoites and SCID2 was inoculated ∼6×10^6^ cryopreserved sporozoites. All horses from the tick-transmitted and sporozoite inoculated groups were monitored for clinical signs of disease and for parasitemia by quantitative real-time PCR, as previously described [32].

### *T. equi*-infected cell cultures

Peripheral blood mononuclear cells were isolated from whole blood of six different immunocompetent adult Arabian horses (HS2-4 and H1-3) and cultured in 24 or 96 well culture plates at 1×10^6^ cells and 2×10^5^ cells per well, respectively. Culture wells were subsequently inoculated with ∼0.1–3.0×10^6^ sporozoites of *T. equi* sporozoites (sporozoite isolation as described above). Cells were sampled for cytospin preparation and Diff-Quick staining to monitor infection by light microscopy. SCID foal PBMC were infected similarly; however, cryopreserved SCID foal PBMC from the previous year were used due to the difficulty of coordinating tick rearing experiments with the availability of SCID foals.

### Flow cytometry and IFA

Infected and uninfected immunocompetent PBMC were harvested from culture wells 9–14 DPI and 5×10^5^ cells were surface labeled with one of three equine leukocyte-specific murine monoclonal antibodies (mAb) (Table 1), including anti-IgM (B lymphocyte), anti-CD3 (T lymphocyte), and anti-CD172a (monocyte/macrophage) and secondarily labeled with goat anti-mouse polyclonal antibody conjugated with PE-Cy5.5 (Caltag Laboratories, Burlingame, CA). The cells were then fixed in BD Cytofix/Cytoperm (BD Bioscience, Mountain View, CA, USA) and internally labeled with mAb specific for equine merozoite antigen 1 and 2 (anti-EMA 1/2) or an IgG1 isotype control mAb (ColiS69A) (Table 1). In previous studies we have demonstrated that EMA 1/2 is expressed by sporoblasts/sporozoites in tick salivary glands [33] and by intraleukocyte schizonts [5]. The anti-EMA 1/2 and isotype control mAb were primarily labeled with Alexa Flour 488 in house as per the manufacturer's protocol (Mouse IgG Labeling Kits, Invitrogen, Carlsbad, CA). A FACSort flow cytometer equipped with a Macintosh computer and Cell Quest software (Becton Dickinson Immunocytometry Systems, San Jose, CA) was used to collect flow cytometry data. Cells not used in flow cytometry were cytospun onto glass slides, dried, and cover slipped with SlowFade Gold antifade reagent with DAPI (Invitrogen) for IFA. IFA images were collected with a Zeiss LCM 510 META laser scanning confocal microscope (Carl Zeiss MicroImaging, Inc., Jena, Germany) using the multi-track feature. Images for control slides and experimental slides were scanned at the same exposure settings. To improve image resolution, the contrast and brightness was slightly altered for all images equally.

**Table 1:** Monoclonal antibodies used.

mAb	Specificity	Working concentration
**1.9/3.2 ** [**48**]	**IgM**	**5 µg/ml**
**F6G ** [**49**]	**CD3**	**20 µg/ml**
**HB88A ** [**50**]	**CD2**	**5 µg/ml**
**HT14A ** [**51**]	**CD8**	**5 µg/ml**
**HB61A ** [**51**]	**CD4**	**5 µg/ml**
**DH59B ** [**50**]	**CD172a**	**5 µg/ml**
**36/133.97 (EMA 1/2) ** [33], [52]	**EMA 1 and EMA 2**	**10 µg/ml**
**ColiS69A ** [53]	***Escherichia coli***	**10 µg/ml**

PBMC were isolated from whole blood of SCID1, SCID2, and Foal1 for leukocyte phenotyping and to detect intracytoplasmic *T. equi* antigen *ex vivo*. The expression of CD3, IgM, CD2, CD8, CD4, and CD172a on SCID and immunocompetent PBMC was assessed by flow cytometry as described above, using the murine anti-equine mAbs (Table 1). All antibodies that labeled greater than 2% of SCID foal PBMC (anti-CD2, anti-CD8, anti-CD4, anti-CD172a) were used to surface label cells prior to performing internal labeling with anti-EMA 1/2 (as described above).

### Necropsy

All SCID foals were humanely euthanized on day 4 or 5 of acute parasitemia. At necropsy the following tissues were examined for gross lesions and collected for histopathology: brain, choroid plexus, pinna, skin, heart, lungs, liver, tracheobronchial lymph node, thymus, mediastinal lymph node, liver, hepatic lymph node, splenic lymph node, spleen, stomach, small intestine, adrenal gland, kidney, mesenteric lymph node, peripheral lymph nodes, and bone marrow. All tissues were fixed in 10% neutral buffered formalin for 72 hr, paraffin embedded, sectioned at 5 µm, and stained with Giemsa tissue stain.

### Statistics

Levels of peak parasitemia, days to parasitemia, and days to peak parasitemia were compared using unpaired t-tests with Welch correction for unequal variances when appropriate. All statistical analyses were performed using GraphPad Prism 5.01 (GraphPad Software, San Diego, CA) and a significance level (α) of 0.05.

## Results

### Intravenous inoculation of *Theileria equi* sporozoites caused acute infection in immunocompetent horses, comparable to vector transmission

**Figure 1:**
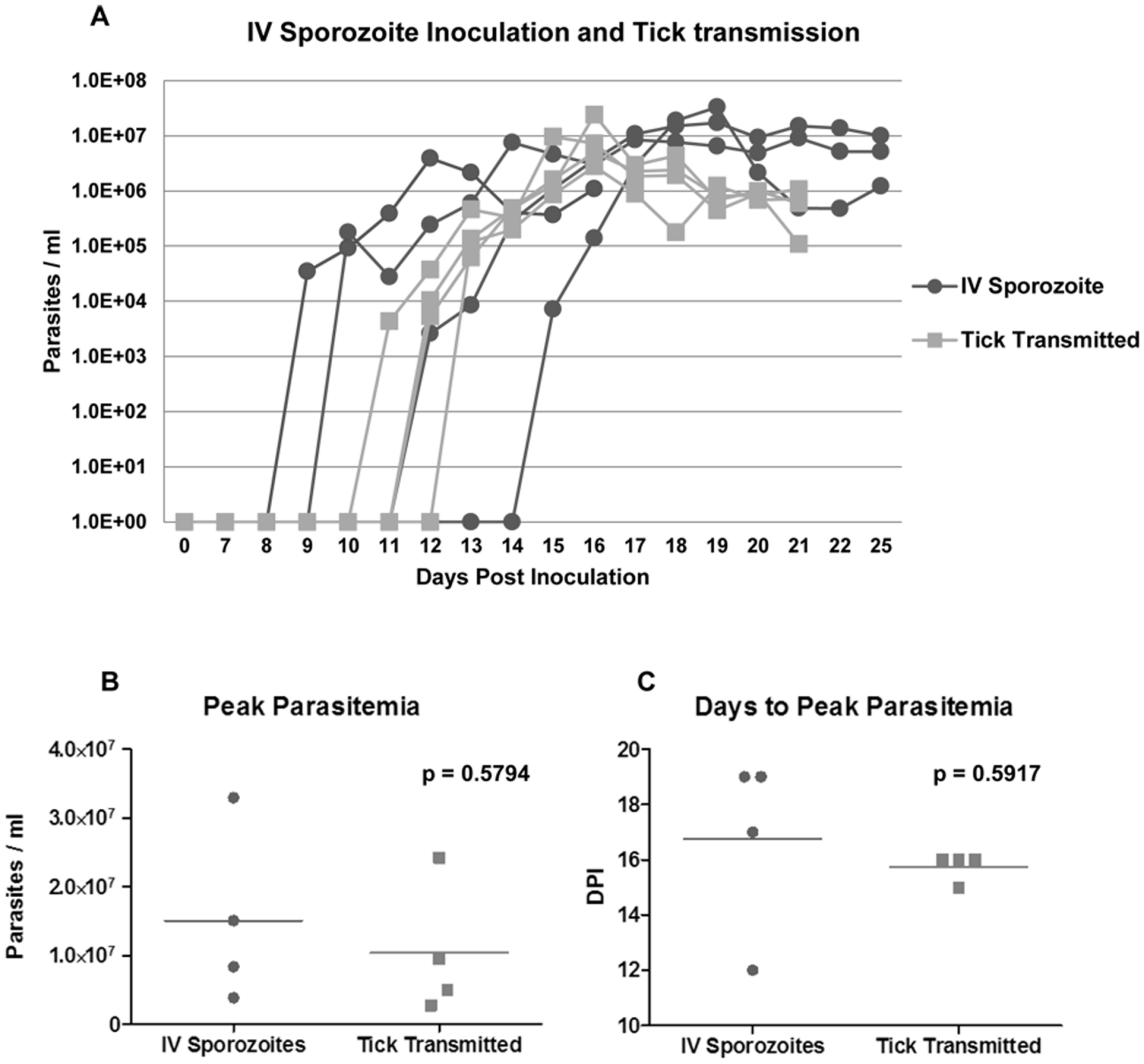
Acute parasitemia after IV sporozoite inoculation and tick-transmission. A. Course of parasitemia (parasites/ml whole blood) for horses infected with *T. equi* sporozoite (∼1×10^5^–10^6^) by intravenous inoculation or by tick-transmission was assessed by quantitative real time PCR. Levels of peak parasitemia (B) and days to peak parasitemia (C) between groups were compared using a t test with α = 0.05. DPI, days post inoculation.

To develop a controlled sporozoite challenge protocol, the course of acute infection caused by intravenous sporozoite inoculation was first compared to that of tick-transmission in immunocompetent horses. Four horses (HS1-4) were inoculated with fresh *T. equi*-infected tick salivary gland homogenates by intravenous injection, while four additional horses (HT1-4) were infected by tick-transmission. In the sporozoite inoculated group, parasitemia was detectable by real time PCR 9–15 days post inoculation (DPI), with peak parasitemia of 10^6^–10^7^ parasites/ml whole blood occurring 12–19 DPI (Fig. 1A). Parasitemia was first detectable in the tick infected group 10–12 DPI, with peak parasitemia of 10^6^–10^7^ parasites/ml whole blood occurring 14–15 DPI. No significant differences were observed in the levels of peak parasitemia or the number of days to peak parasitemia between the inoculation and tick-transmission groups (Fig. 1B, C). Thus, the course of acute infection caused by intravenous sporozoite inoculation was similar to that of tick-transmission.

### *T. equi* Florida strain sporozoites infected peripheral blood mononuclear cells in vitro

**Figure 2:**
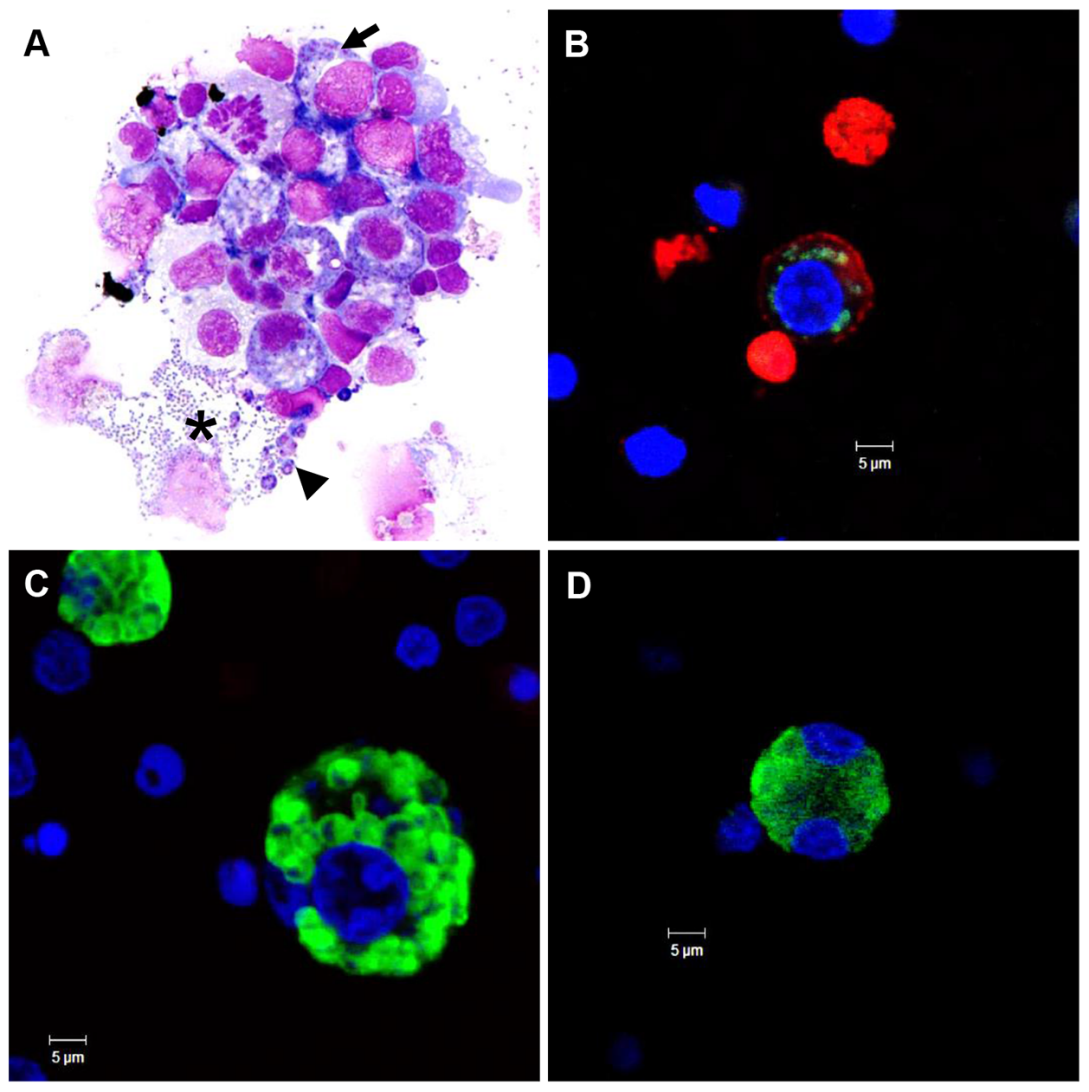
In vitro PBMC infection with *T. equi* sporozoites. *T. equi* (Florida strain) infected PBMC day 9-11 in culture; Diff-Quick stained, cytospin preparation (A), and IFA (B, C, and D). (A) Schizont-infected cells have few to abundant, ∼0.5–1.0 µm diameter, purple, intracytoplasmic parasite nuclei (arrow). Adjacent to schizont-infected cells there are several extracellular merozoites (asterix), schizonts (arrowhead), and cellular debris. (B, C and D) IFA images of cells containing different schizont forms are labeled with anti-EMA 1/2 (green). (B) Infected cell is surface labeled with anti-IgM mAb (B lymphocyte). (C, D) Infected cells did not express detectable leukocyte specific surface markers. Nuclei are stained blue with DAPI.

Fresh PBMC from six different immunocompetent horses were infected *in vitro* with *T. equi*-infected tick salivary gland homogenates (∼0.1–3.0×10^6^ sporozoites) to characterize leukocyte infection in culture. Infection was first detectable by light microscopy in a subpopulation of PBMC between 3–6 DPI and viable schizont-infected cells could be identified up to 45 DPI (data not shown). In contrast, uninfected control PBMC only survived in culture for 12–15 days (data not shown). Intracellular macroschizonts and extracellular merozoites were detectable by light microscopy between 6-12 DPI (representative photomicrograph, Fig. 2A). Schizont-infected cells were identified by the presence of few to abundant, small (∼0.5 to 1.0 µm), purple, intracytoplasmic parasite nuclei. Schizont-infected cells undergoing mitosis were extremely rare (i.e. approximately one observed in >50 Diff-Quick stained cytospin preparations). Debris from ruptured cells, extracellular merozoites, and extracellular schizonts were commonly identified adjacent to intact cells (Fig. 2A). *T. equi* infection was confirmed by IFA using anti-EMA 1/2 mAb. The immunoreactivity of intracytoplasmic schizonts varied from multifocal and discrete to diffuse, and was consistent with the morphology and distribution of the parasites observed with light microscopy (Fig. 2B, C, D). Inoculation of erythrocyte cultures with ∼1×10^6^ fresh *T. equi* sporozoites did not result in infection (data not shown). These findings are consistent with previous reports and demonstrate the capacity of *T. equi* Florida strain to infect leukocytes [3], [34].

### *T. equi* schizont-infected PBMC expressed macrophage, B lymphocyte, or T lymphocyte specific surface markers *in vitro*

**Figure 3:**
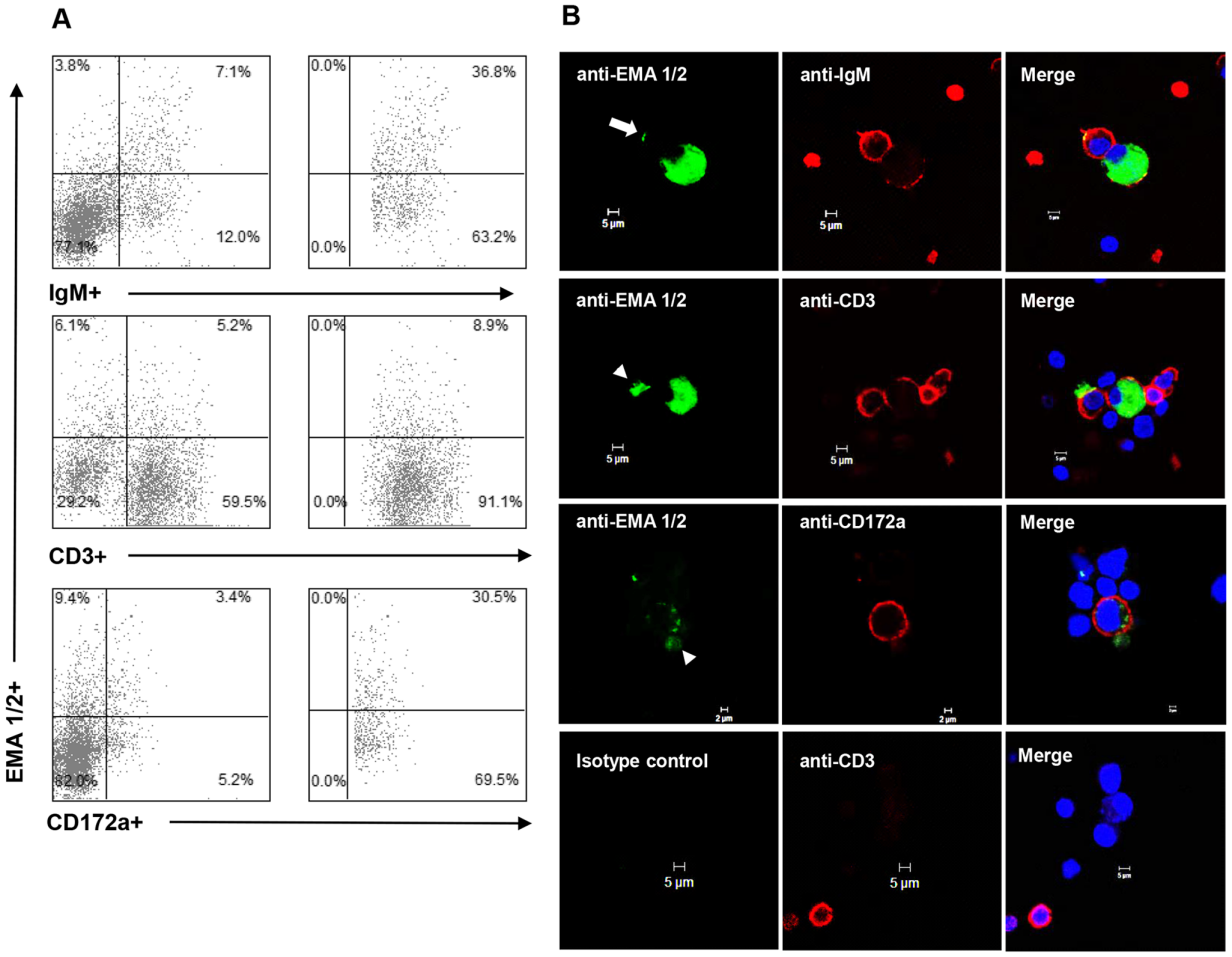
Immunophenotype of schizont-infected PBMC *in vitro*. Flow cytometric (A) and IFA (B) analysis of schizont-infected cells *in vitro*. (A) Representative flow cytometric data for infect horse H2. Left column: percent of total PBMC dual labeled with leukocyte specific mAbs and *T. equi* specific mAb (anti-EMA 1/2). Right column: percent infected cells determined on IgM^+^ (B lymphocyte), CD3^+^ (T lymphocyte), or CD172a^+^ (macrophage) gated leukocytes. (B) IFA images of schizont-infected cells dual-labeled with one of the three leukocyte specific mAb and anti-EMA 1/2. The nuclei of all cells were stained blue with DAPI. The anti-EMA 1/2 mAb either formed diffuse signal throughout the cytoplasm of infected cells or discrete signal along the surface of intracytoplasmic macroschizonts. Rare cells had punctate foci of EMA 1/2^+^ immunoreactivity along their outer margin (top left panel; arrow). The space adjacent to cells multifocally contained scant, irregular to round, immunoreactive debris (left panels of second and third row; arrowhead). The anti-IgM, anti-CD3, and anti-CD172a mAb formed punctate to diffuse signal along the outer surface of infected cells. Labeling with an isotype control for the anti-EMA 1/2 mAb did not form any detectable signal (representative data shown in the bottom panels of B).

To determine the phenotype of schizont-infected leukocytes, whole PBMC from three immunocompetent horses (H1-3) were infected with *T. equi* sporozoites *in vitro* and analyzed by flow cytometry and IFA (Fig. 3). A panel of three mAb [anti-IgM (B lymphocyte), anti-CD3 (T lymphocyte), or anti-CD172a (macrophage)] were used to surface label the cells, and intracellular staining with a second mAb specific for equine merozoite antigen 1 and 2 (anti-EMA 1/2) was used to detect infection. H1 cells were used in a pilot experiment to optimize immunolabling and determine the time point (DPI) when both surface and internal labeling was readily detectable by flow cytometry and IFA (data not shown). Based on the findings of this experiment, phenotyping of H2 and H3 infected cells was performed 10–11 DPI. In H2 and H3 cultures analyzed by flow cytometry, a subset of each leukocyte phenotype was dual labeled with anti-EMA 1/2 (Fig. 3A). Further analysis of each leukocyte sub-population demonstrated that a relatively high proportion of the B lymphocytes (range: 34.5% to 36.8%) and macrophages (range: 31.5% to 31.0%) were infected, as compared to the low proportion of T lymphocytes (range: 4.5% to 8.9%) (Fig. 3A), suggesting that *T. equi* preferentially infects B lymphocytes and macrophages. Immunofluorescence microscopy verified that the vast majority of dual labeled cells were infected with schizonts; however, there were rare cells that had punctate foci of immunoreactivity with the EMA 1/2 mAb along their outer margin (Fig. 3B, arrow) and the space adjacent to cells multifocally contained scant, irregular to round, immunoreactive debris (Fig. 3B, arrowhead). The presence of this extracellular EMA 1/2^+^ material is consistent with the extracellular merozoites and schizonts identified by light microscopy (Fig. 2A). Overall, the signal generated by the leukocyte-specific monoclonal antibodies was unevenly distributed and less abundant on infected cells as compared to uninfected cells (Fig. 3B).

### Intravenous sporozoite inoculation of SCID foals caused patent merozoite parasitemia

**Figure 4:**
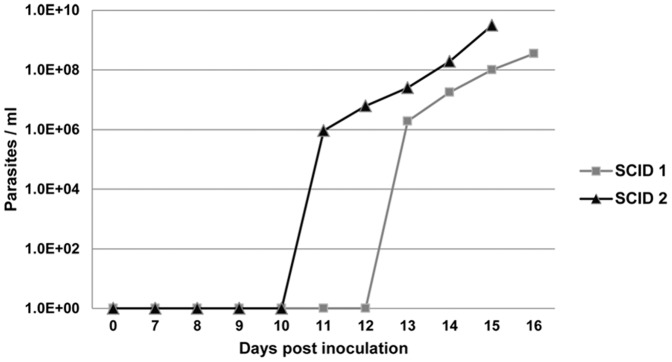
SCID foal infection with cryopreserved sporozoites. Course of parasitemia for SCID foals infected with sporozoites by intravenous inoculation. SCID1 = 4×10^6^; SCID2 = 6×10^6^ sporozoites.

**Figure 5:**
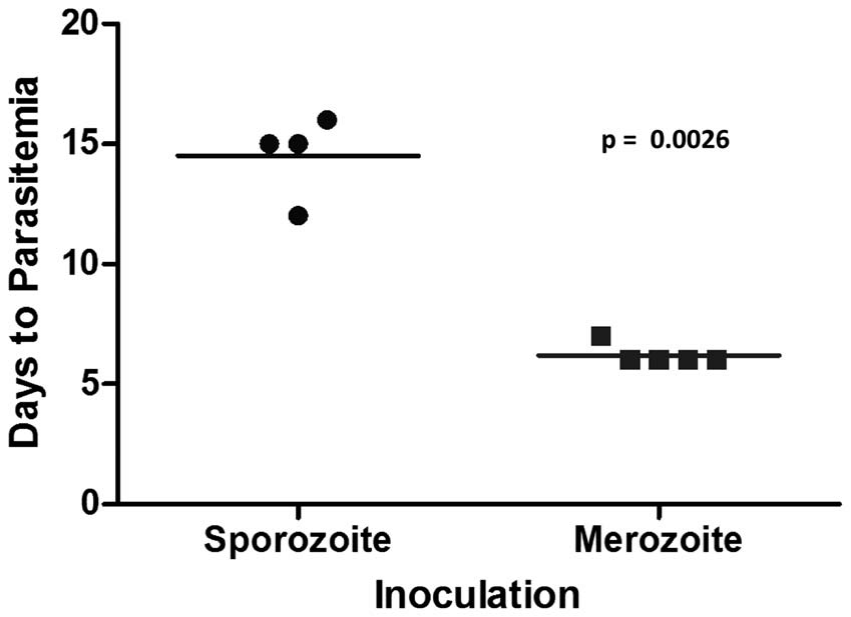
Prepatent period: sporozoite vs. merozoite inoculation. Days to first detectable parasitemia for immunocompetent and SCID foals inoculated intravenously with sporozoites was compared with that for historical SCID controls inoculated with merozoite-parasitized erythrocyte stabilates [35], [36], using a t test (α = 0.05).

SCID foals were infected with cryopreserved *T. equi* sporozoites by intravenous inoculation to determine if B lymphocytes and/or T lymphocytes were required for schizont differentiation *in vivo*. In the first experimental inoculation, one SCID foal (SCID1), one age-matched immunocompetent foal (Foal1), and two adult horses, were inoculated with cryopreserved tick salivary gland homogenates containing ∼1×10^6^ sporozoites (determined by real-time PCR on a cohort of infected ticks). One of the adult horses and Foal1 developed patent merozoite parasitemia by 9 and 21 DPI, respectively. SCID1 did not become parasitemic and remained PCR negative until the end of the experimental period (28 DPI). The second adult horse similarly remained PCR negative throughout the experimental period (74 DPI), suggesting that after cryopreservation, a dose of 1×10^6^ sporozoites was at the threshold of the infectious dose. To rule out the possibility that the lack of infection in SCID1 was due to innate resistance caused by lymphocyte deficiency, SCID1 was re-inoculated with 4×10^6^ cryopreserved sporozoites and a second SCID foal (SCID2) was inoculated with 6×10^6^ cryopreserved sporozoites. Both SCID1 and SCID2 became PCR positive 13 and 11 DPI, respectively, and developed uncontrolled parasitemia (Fig. 4). These findings demonstrated that horses lacking functional T and B lymphocytes could be infected with *T. equi* sporozoites. Finally, the course of infection in SCID and normal horses caused by sporozoite inoculation was compared to that of merozoite inoculation performed in previous published [35], [36] and unpublished experiments. This analysis demonstrated that the prepatent period of sporozoite infection was 6–10 days longer than merozoite infection (Fig. 5). This observation was consistent with the time required to complete schizogony in culture and provides additional support for intraleukocyte schizont development in SCID foals.

### *T. equi* Florida strain sporozoites infected SCID foal peripheral blood mononuclear cells *in vitro*

Although it is well known that SCID foals lack B and T lymphocytes [23]–[25], [27]–[29], [37], flow cytometric analysis was performed for confirmation on SCID1 and SCID2 PBMC using a panel of six mAb against equine B lymphocytes (anti-IgM), T lymphocytes (anti-CD3, CD2, CD8, and CD4), and macrophages (anti-CD172a). In both SCID1 and SCID2, less than 1% of the PBMC analyzed were identified as B or T lymphocytes (data not shown). Approximately 5% of PBMC were CD2^+^/CD3^−^, consistent with a natural killer (NK) cell phenotype [38], [39]. A similar proportion of SCID PBMC were CD8^+^ (∼4%) or CD4^+^ (∼6%). Given the lack of CD3^+^ cells, CD8^+^ and CD4^+^ cells were interpreted to be NK cells and monocytes/macrophages, respectively. Greater than 90% of the analyzed PBMC were CD172a^+^, indicating that they were monocytes/macrophages (data not shown). Although the anti-CD172a mAb also identifies equine granulocytes [40], the ficoll hypaque PBMC isolation protocol (which removes the majority of granulocytes) together with the lack of internal complexity in the labeled cells (data not shown), provided further evidence that they were monocytes/macrophages. Overall, these data confirmed the SCID phenotype and indicated that the establishment of *T. equi* infection in SCID1 and SCID2 did not involve B or T lymphocytes.

Next, cryopreserved SCID foal PBMC were inoculated with 4×10^5^ fresh *T. equi* sporozoites *in vitro* to confirm infection of SCID leukocytes. Prior to cryopreservation of these PBMC, flow cytometric analysis was performed as above which confirmed the same phenotypic distribution of cells, including the lack of B and T lymphocytes (data not shown). On day 6 post inoculation, schizont-infected mononuclear cells were identified by light microscopy in the cultures from both SCID foals (data not shown). Due to the poor survival of cryopreserved SCID PBMC in culture and the paucity of schizont-infected cells, further analysis to determine the phenotype of these schizont-infected cells was not possible.

### Persistent leukocyte infection was not detected in SCID foals

**Figure 6:**
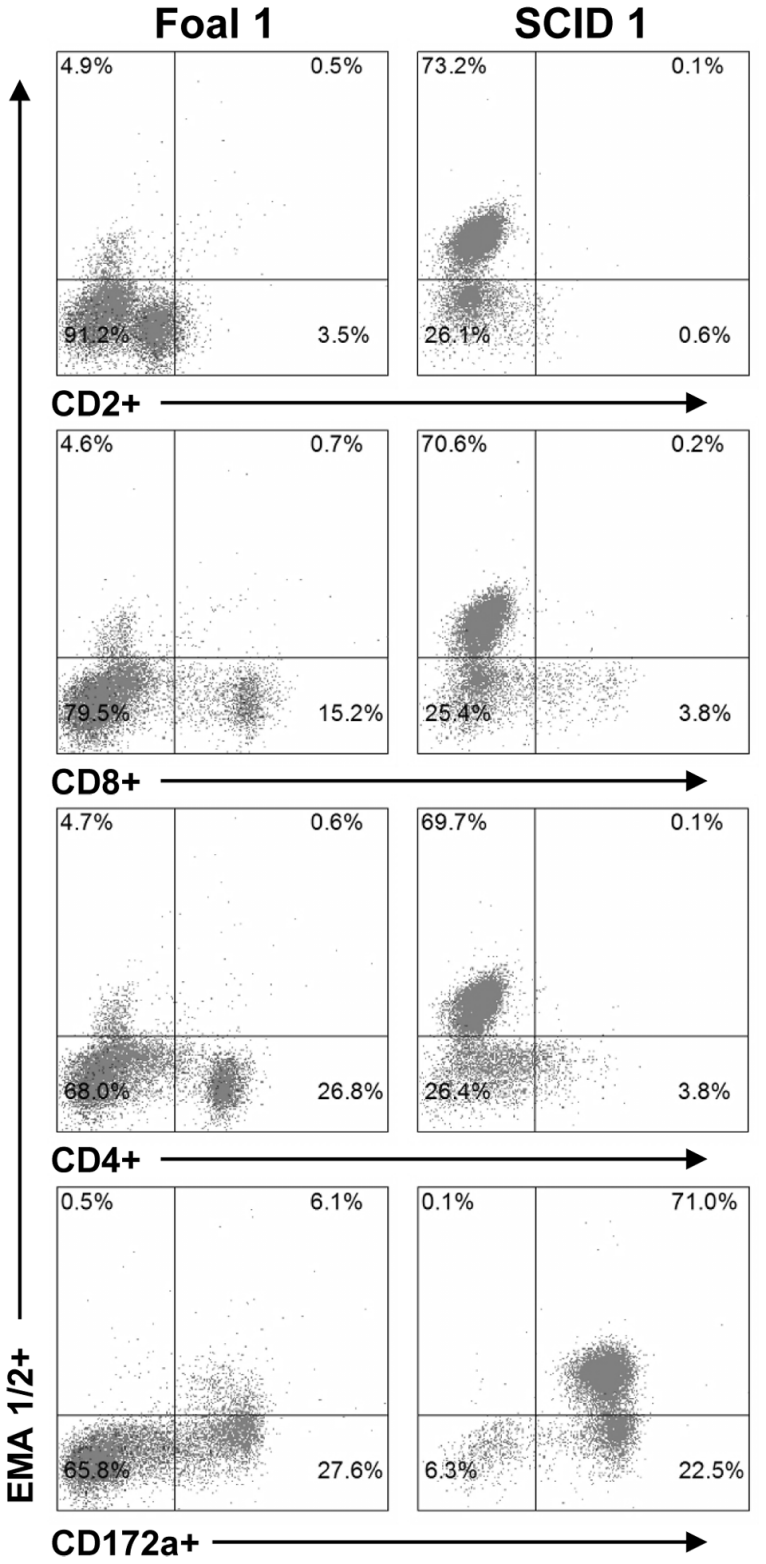
Immunophenotype of immunocompetent and SCID foal PBMC containing *T. equi* antigen *ex vivo*. PBMC from Foal1 (48 DPI) and SCID1 (15 DPI) were analyzed post-infection by flow cytometry to determine the percentage of CD2^+^, CD8^+^, CD4^+^, and CD172a^+^ cells that contained *T. equi* antigen (EMA 1/2).

To determine if schizont-infected leukocytes persist in the absence of an adaptive immune response, tissue samples and peripheral blood from SCID1 and SCID2 were assessed for the presence of schizont-infected cells. Prior to the termination of the experiment, PBMC were isolated from SCID1 (15 DPI) and immunocompetent Foal1 (48 DPI) and analyzed by flow cytometry as above with the addition of intracellular staining using anti-EMA 1/2 to identify cells containing *T. equi* antigen (Fig. 6). Monocytes/macrophages were the only cells that contained equine merozoite antigen (EMA). Cytospin preparations of fresh PBMC from SCID1 and SCID2 consisted of abundant histiocytic round cells, with no detectable schizont-infected cells (data not shown). This finding suggested that the antigen detected in monocytes/macrophages by flow cytometry was due to phagocytosis of merozoite infected erythrocytes and not due to persistent schizont infection of monocytes. Postmortem tissue samples were collected from SCID1, SCID2, and Foal1 for histologic examination following Giemsa staining. No schizont-infected cells were identified in any of these tissues from any animal (data not shown).

## Discussion

In previous studies, *T. equi* schizont-infected cells have been characterized *in vitro* and *ex vivo* as lymphocytes based on their microscopic and ultrastructural morphology [4], [41], [42]. The subtle differences between lymphocyte and monocyte/macrophage morphology (e.g. amount of cytoplasm, nuclear shape) are, however, distorted by intracytoplasmic schizont development and precludes determining the specific identity of infected cells. The present study was designed to resolve the phenotype of schizont-infected cells *in vitro* and determine if lymphocytes were required to establish infection *in vivo*. Flow cytometric analysis of PBMC infected *in vitro* demonstrated that *T. equi* infected B lymphocytes, T lymphocytes, and monocyte/macrophages. Subsequent immunefluorescent microscopic analysis confirmed that the vast majority of cells labeled with *T. equi* antigen specific mAb contained schizonts within their cytoplasm; however, there were rare cells that had small, punctate foci of immunoreactivity with the EMA 1/2 mAb along the outer margin of the cell. Since all cultures examined contained merozoites released from infected leukocytes, it could not be determined if these small foci of immunoreactivity were sporozoites arrested during initial invasion, merozoites attached to the host cell membrane, or simply immunoreactive debris within the culture. Regardless, these findings demonstrate for the first time that the host cell range of *T. equi* extends to include both lymphocytes and monocyte/macrophages, making it the most promiscuous among related *Theileria* spp. (*T. annulata and T. parva*).

The specific host surface molecule(s) recognized and bound by Theileria sporozoites during leukocyte invasion is not defined; however, MHC class I molecules have been shown to be essential for *T. parva* sporozoite host-cell recognition and binding [43]. The fact that MHC class I molecules are ubiquitously expressed on nucleated cells suggests that other host molecules must also be involved to facilitate a selective and specific interaction. The observation that antibodies reactive with a number of lymphocyte cell surface molecules also block *T. parva* sporozoite entry supports this hypothesis [44]. The relatively broad host-cell range of *T. equi* characterized in the present study suggests that the specific molecules recognized by *T. equi* sporozoites are common to lymphocytes and macrophages; thus, future work to characterize the host cell receptor recognized by *T. equi* sporozoites should focus on shared molecules.

Interestingly, both *T. parva* and *T. annulata* alter the expression of surface and non-surface associated host molecules (e.g. surface immunoglobulin, T cell markers, MHC class I molecules, pro-inflammatory cytokines) [13], [20], [21], [45]–[47]. In the IFA performed in the present study, IgM, CD3, and CD172a labeling was reduced and unevenly distributed on the surface of infected cells as compared to uninfected cells. This finding suggests that *T. equi* alters the expression of cell surface molecules similar to *T. annulata* and *T. parva*; however, additional work is required to more specifically and quantitatively assess the impact *T. equi* has on the expression of host surface and non-surface associated molecules.

The finding that *T. equi* leukocyte tropism overlaps with both *T. annulata* and *T. parva* is of interest because it suggests that the protective immune responses directed against each of the latter *Theileria* spp. schizont stages (i.e. CTL and cytostatic macrophages) would also be important for protection against *T. equi*. It is known that horses infected with *T. equi* are resistant to disease when they are re-exposed to the parasite [1], but at present the mechanism of resistance is unknown. Antigens that elicit immune responses directed against the pre-erythrocytic stages of *T. equi* represent attractive vaccine candidates because they have the potential to control infection prior to the pathogenic and tick-transmissible blood stage. The apparent transient nature of *T. equi* schizonts suggests that these responses may be more difficult to elicit in the horse; however, *Bos indicus* cattle infected with *T. parva*-infected B lymphocyte lines develop a similarly transient and self-limiting schizont parasitosis, but are still protected against lethal challenge [19].

To evaluate the leukocyte tropism of *T. equi in vivo* an intravenous sporozoite challenge model was developed to more precisely control the inoculation dose and eliminate potential problems associated with tick-transmission in SCID foals (i.e., secondary bacterial infection at site of tick attachment, unexpected tick mortality during transmission feeding, and physiologic stress caused by feeding ticks on young horses). In immunocompetent horses there was no statistically significant difference between the course of parasitemia caused by tick-transmission versus intravenous sporozoite inoculation. However, the time to peak parasitemia was more variable in the sporozoite inoculated group. Although the cause for this greater variability is not known, it may have been related to a difference in the sporozoite dose provided by a single intravenous inoculation versus that of a ten day tick-transmission. To test hypotheses directly related to infection kinetics in the future, it will be important to determine how dose and other factors (e.g., route of infection) influence the time to peak parasitemia. For the present study, intravenous sporozoite inoculation allowed testing of the stated hypothesis and avoided the aforementioned problems with tick-transmission.

Establishing *T. equi* infection in two SCID foals by sporozoite inoculation confirmed that the leukocyte tropism of *T. equi* extends beyond T and B lymphocytes, and also demonstrated the utility of this model for *T. equi* immunology research. Specifically, SCID foals could be used to study innate immune responses *in vivo* in the absence of adaptive immunity. Furthermore, it should be possible to directly determine the correlates of protective adaptive immunity against *T. equi* pre-erythrocytic stages by adoptive transfer of antigen specific T lymphocytes or by passive antibody transfer experiments [35]. Since the phenotype of schizont-infected cells could impact pathogenesis and strain virulence, it would be important to fully characterize the phenotype of schizont-infected SCID leukocytes. Based on the phenotypic analysis performed here, it was concluded that monocyte/macrophages comprised the cell type most likely infected in SCID foals. However, since SCID foals also possess NK cells [38], [39], the possibility that NK cells were also infected *in vivo* cannot be ruled out.

Although the primary reason for performing the SCID foal experiments was to establish the necessity of B and T lymphocytes in *T. equi* pathogenesis, the secondary goal was to determine whether the absence of adaptive immunity resulted in a prolonged course of leukocyte infection. Since merozoite parasitemia is uncontrolled and rapidly fatal in SCID foals [35], post-infection flow cytometric analysis could not be performed beyond the acute stage of disease (i.e., 15 DPI). This analysis demonstrated that monocyte/macrophages were the only cells that contained the EMA 1/2 antigen. When these PBMC were examined by light microscopy however, no schizont-infected cells were identified. This suggested that the antigen detected within these cells was due to the phagocytosis of parasitized erythrocytes and not persistent schizont infection. Histologic examination of postmortem tissues similarly failed to identify schizont-infected cells, providing additional evidence that the schizont stage does not persist *in vivo*. This finding is consistent with the apparent transient nature of the schizont-infected cells in immunocompetent horses and in the cultures of the present study.

In contrast to the results reported here, others have described *T. equi* schizonts transforming PBMC into lymphoblastoid forms that proliferate for up to 5 months in culture [4], [42]. Unfortunately, the previously cited studies were largely descriptive and did not report the specific strain of *T. equi* used; therefore, we cannot provide meaningful speculation regarding the likely reason for these differing results. The lack of leukocyte transformation with the strain of *T. equi* used herein (Florida strain) is consistent with the genomic analysis of this strain, which did not identify homologs to the putative Theileria host cell transforming genes [5].

In summary, these data provide for the first time a comprehensive and specific analysis of *T. equi* leukocyte tropism, and demonstrate that the *T. equi* host cell range extends beyond lymphocytes. The finding that the *T. equi* host cell range includes monocytes/macrophages establishes a closer phenotypic relationship with *T. annulata*, and justifies the investigation of adaptive and innate immune responses that are important against Tropical Theileriosis (e.g. cytostatic macrophages). Importantly, the observation that SCID foals can become infected following inoculation with *T. equi* pre-erythrocytic stages establishes an *in vivo* model for dissecting mechanisms of both innate and adaptive of immunity directed against sporozoites and schizonts. Finally, this work provides the basis for future research to determine the mechanism of leukocyte recognition and invasion, characterize the host cell functions altered by parasite infection, and investigating leukocyte tropism as it relates to other phenotypic traits.
